# Evaluation of autoantibodies to common and neuronal cell antigens in Chronic Fatigue Syndrome

**DOI:** 10.1186/1740-2557-2-5

**Published:** 2005-05-25

**Authors:** Suzanne D Vernon, William C Reeves

**Affiliations:** 1 Division of Viral and Rickettsial Diseases, National Center for Infectious Diseases, Centers for Disease Control and Prevention, Atlanta, Georgia 30333, USA

**Keywords:** chronic fatigue syndrome, CFS, antibodies, autoantibodies

## Abstract

People with chronic fatigue syndrome (CFS) suffer from multiple symptoms including fatigue, impaired memory and concentration, unrefreshing sleep and musculoskeletal pain. The exact causes of CFS are not known, but the symptom complex resembles that of several diseases that affect the immune system and autoantibodies may provide clues to the various etiologies of CFS. We used ELISA, immunoblot and commercially available assays to test serum from subjects enrolled in a physician-based surveillance study conducted in Atlanta, Georgia and a population-based study in Wichita, Kansas for a number of common autoantibodies and antibodies to neuron specific antigens. Subsets of those with CFS had higher rates of antibodies to microtubule-associated protein 2 (MAP2) (p = 0.03) and ssDNA (p = 0.04). There was no evidence of higher rates for several common nuclear and cellular antigens in people with CFS. Autoantibodies to specific host cell antigens may be a useful approach for identifying subsets of people with CFS, identify biomarkers, and provide clues to CFS etiologies.

## Background

Chronic fatigue syndrome (CFS) is defined as persistent or relapsing fatigue that has occurred for at least 6 months, is not alleviated by rest, and causes substantial reduction in activities. The fatigue cannot be explained by medical or psychiatric conditions and must be accompanied by at least 4 of 8 specified symptoms (unusual post exertional fatigue, impaired memory or concentration, unrefreshing sleep, headaches, muscle pain, joint pain, sore throat, and tender cervical nodes) [1]. There is considerable discrepancy in results between studies from different institutions; so as yet, there are no characteristic signs or laboratory markers of CFS and its pathophysiology has not been elucidated [2].

This lack of diagnostic signs or laboratory markers notwithstanding, many manifestations of CFS resemble those of musculoskeletal and infectious diseases [3]. In large part, the illnesses caused by these diseases reflect immune system activation and there is evidence for immune system dysfunction in some cases of CFS. In particular, antinuclear antibodies (ANA) and other common autoantibodies have been evaluated in people with CFS: unfortunately, with variable results. For example, one study found that 52% of tertiary care- CFS referral-patients had antibodies to nuclear envelope antigens [4] while another study found the same ANA antibody rates in both CFS and controls [5]. Recently, investigators reported that antibodies to the human muscarinic cholinergic receptor 1 may provide a biologic explanation for the cognitive impairment observed in people with CFS [6]. This lack of consensus between studies may in large-part reflect recruitment bias associated with studies of persons enrolled from tertiary referral clinics combined with imprecise evaluation of the illness and inadequate or inappropriate control populations.

We had the opportunity to measure the associations of common autoantibodies and autoantibodies to neuronal cell antigens and CFS in two case control studies; one of primary care patients with CFS who were identified by a physician surveillance network; the other a study of people with CFS identified from the community. The physician surveillance study was conducted 1988 through 1993 in Atlanta, Georgia [7] and the community study 1997 through 2000 and identified subjects with CFS from the general population of Wichita, Kansas [8].

Both studies rigorously classified people as CFS and controls in both studies were enrolled to represent the general population and matched to cases by sex, race, and age [9,10]. The hypothesis of the present study is that the appearance of cell-specific autoimmune antibodies may define subsets of CFS and give clues to the etiology and pathogenesis and may help to explain the neurocognitive symptoms experienced by CFS patients. Secondarily, we wished to evaluate the extent to which patients with CFS who were receiving primary medical care treatment for CFS were similar to people with CFS in the community.

## Methods

### Study Subjects and samples

Both studies adhered to human experimentation guidelines of the U.S. Department of Health and Human Services and the Helsinki Declaration. The Centers for Disease Control and Prevention (CDC) Institutional Review Board approved study protocols. All participants were volunteers who gave informed consent.

#### Physician surveillance study

Between 1988 and 1993, the CDC conducted a physician surveillance survey for CFS in primary care patients from Reno, Nevada, Wichita, Kansas, Grand Rapids, Michigan, and Atlanta, Georgia [7]. Patients were classified as CFS according to the 1988 case definition [11]. In 1992, we conducted a case control study of CFS patients and controls in Atlanta by recruiting patients from physician surveillance and sex, race, age matched non fatigued controls identified in the general Atlanta population [9,10]. The case control study classified patients as CFS according to the study collected information concerning several risk factors and blood to measure associations between CFS and laboratory markers. The present study used remaining archived serum samples from 22 CFS patients and 34 age and sex matched controls. All CFS patients met criteria of the current CFS research case definition [1]

#### Population study participants

Between 1997 and 2000, CDC conducted surveillance of CFS in the general population of Wichita, Kansas [8]. Briefly, the study involved random digit dial surveys to identify people with CFS-like illness and clinically evaluated and classified them according to criteria of the 1994 CFS research case definition [1]. Only 16% of those identified with CFS had been diagnosed or treated for CFS by a physician [12]. The present study used archived serum samples from 37 subjects with CFS and a 57 non-fatigued control subjects

#### Blood samples

Both the physician surveillance and population study collected blood in BD Vacutainer Serum tubes. The samples were shipped by overnight courier to CDC where they were dispensed into 0.5 ml aliquots and stored at -80°C until testing.

### Reagents and Assays

Commercially available kits were used for antibodies to ubiquitous nuclear and cellular autoantigens including dsDNA, ssDNA, Sm, U1-RNP, SS-A/Ro, SS-B/La, Scl-70, and Centromere. Immunoassays were purchased from Helix Diagnostics (West Sacramento, CA) and reagents for western blots were purchased from Diagnostic Products Corporation (Los Angeles, CA). Purified Histone H3, and Histone H4 were purchased from Sigma (St. Louis, MO). and used in ELISA assays that were developed at Scripps. Conventional immunofluorescent antinuclear antibodies and rheumatoid factor tests were performed as described previously [13]. Preparations of microtubule-associated protein 2 (MAP2) and neurofilament triplet (NFT) proteins were purchased from Sigma (St. Louis, MO). The commercially available ELISA assays were performed according to the manufacturers instructions. The ELISA and western blot assays for the neuronal antigens were developed at Scripps and were performed as previously described [14].

### Statistical Analysis

Because the subjects are derived from studies that are distinct in design and geographic location, each study was analyzed separately. The distribution of autoantibodies between CFS and non-fatigued controls was compared by Fisher exact probability test. To derive an estimate of confidence, stratified groups were compared by the non-parametric chi square test. To determine associations, subjects were stratified by sex, age, and CFS for all MAP2, NFT and ssDNA. The association of autoantibodes in CFS subjects was compared by grouping by sex, age, age at illness onset, and duration of illness. CFS subjects were stratified by sex, age (<40 years, 40–40 years, >50 years), onset type (gradual versus sudden) and duration of illness (<5 years, >5 years) to determine whether an association with autoantibodies existed.

## Results

Although women predominated in both study groups other demographic and clinical characteristics differed and reflected basic differences between patients with CFS who obtain medical care and those in the general population (most of whom have not seen a physician) (Table 1). Of note, CFS cases from physician surveillance were somewhat younger than those identified in the population (mean 39 and 46 years, respectively) and controls were similarly different: those recruited in the physician study had been ill about half as long as those in the community (69 and 128 months, respectively) and were more likely to report sudden onset CFS (36.4%) than those in the general population with CFS (13.5%).

**Table 1:** Characteristics of subjects evaluated for autoantibodies.

	CFS	Non-Fatigued
Atlanta Case Control		
Subjects (n = 56)	22	34
Female (n = 52)	19	33
Male (n = 4)	3	1
Age Group (yrs)		
18–29 (n = 9)	3	6
30–39 (n = 18)	7	11
40–49 (n = 23)	12	11
50–59 (n = 6)	0	6
Mean Age	39 years	38 years
Mean Age Onset	35 years	
Onset type		
Sudden	8	
Gradual	14	
Mean Illness Duration	69 months	
Number of Subjects Ill		
< 5 years	10	
>5 years	12	
		
Wichita Population		
Subjects (n = 94)	37	57
Female (n = 64)	31	33
Male (n = 30)	6	24
Age Group (yrs)		
18–29 (n = 14)	1	13
30–39 (n = 18)	8	10
40–49 (n = 26)	13	13
50–69 (n = 36)	15	21
Mean Age	46 years	42 years
Mean Age Onset	36 years	
Onset type		
Sudden	5	
Gradual	32	
Mean Illness Duration	128 months	
Number of Subjects Ill		
< 5 years	14	
>5 years	23	

A few CFS subjects in the physician surveillance study aged 18–29 years had antibodies to ssDNA when compared to the same age non-fatigued control group. The mean value for the 3 CFS subjects was 2-fold greater then in the 6 non-fatigued controls (p = 0.038). Among CFS subjects, the 10 who reported being ill for ≤ 5 years had lower levels of autoantibodies to MAP2 (median value of 18, range 12 – 20) compared to the 12 CFS subjects who have been ill for >5 years (median value of 8, range 6 to 10) (p = 0.025). There were no other significant findings in the physician surveillance CFS subjects when stratified by sex or type of illness onset.

In the population-based study, there was a significant difference in the prevalence of autoantibodies to MAP2 between the 30 male subjects (20/30, 67% positive) and the 64 female subjects (19/64, 30% positive) (p = 0.0006). Among the non-fatigued control group, 9 of 33 women (27%) and 19 of 24 (79%) men were positive for antibodies to MAP2 (p = 0.0004). One male CFS subject (16%) was positive for MAP2 antibodies compared to 79% (19/24) male non-fatigued controls (p = 0.04). Among CFS subjects that were ≤ 40 years of age, there was a trend for lower MAP2 antibody levels for those that were ill for ≤ 5 years compared to those ill for >5 years (p = 0.056).

## Discussion

CFS is a complex, debilitating illness, which is characterized by at least 6 months of severe persistent or relapsing fatigue and a group of characteristic but nonspecific symptoms. Despite more than a two decades of extensive research, no diagnostic tests exist, and effective control and prevention remain elusive because the cause and pathophysiology of CFS remain unknown. CFS is clinically similar to several rheumatic autoimmune disorders that can be diagnosed and characterized by autoantibody profiles. For this reason, we conducted an exhaustive evaluation of 11 ubiquitous nuclear and cellular autoantigens in addition to two neuronal specific antigens.

The serum samples tested in this study were collected from a physician surveillance study conducted in Atlanta [15] and a population-based community study in Wichita [8]. The physician surveillance study was conducted over 7 years and used approximately 70% of all primary care physicians in Atlanta. All patients were carefully evaluated for unexplained unwellness and fatigue. The community study was a random digit dial survey of 90,000 people (25% of the Wichita, Kansas population). All CFS cases were medically and psychiatrically evaluated and rigorously classified as CFS, other unexplained unwellness, or medically/psychiatrically explained unwellness. The serum evaluated in this study includes carefully evaluated CFS subjects, matched controls and non-fatigued controls from the community. Therefore, the results from this study should be applicable to similarly designed studies.

Very few studies have evaluated the presence of autoantibodies in people with CFS. Those that have tested for the same autoantibodies report discordant results. Konstantinov et al, [4] found high rates of antinuclear antibodies (ANA) in CFS patients while Skowera et al, [5] found no difference in the rate of ANA between CFS patients and controls. One explanation for these discrepancies could be a technical one where laboratories used different reagents and methods resulting in discordant results. Another explanation could be that the CFS subjects were evaluated differently. Rigor in evaluating CFS patients and applying the case definition [1] is pivotal and undoubtedly accounts for much of the variation. The results presented also show little evidence for autoantibodies to ubiquitous nuclear and cellular autoantibodies.

The findings of this study hint that evaluation of certain autoantibodies may give clues to etiology and ongoing pathology in subsets of CFS subjects. A few of the physician surveillance CFS cases from the youngest age category had autoantibodies to ssDNA. Autoantibodies to ssDNA have been associated with both viral and bacterial infection [16,17]. Interestingly, CFS subjects who describe a sudden onset to their illness often report flu-like illness. The fact that antibodies to ssDNA were detected only in this age group may reflect an immune response to infection commonly affecting this age group, such as infectious mononucleosis from Epstein Barr Virus infection.

There was a higher prevalence of autoantibodies to MAP2 in the non-fatigued men in the community study compared to the non-fatigued women. The significance of this finding is not known but highlights the importance of carefully stratifying and controlling for factors that could affect interpretation of results. There did seem to be a slight association of MAP2 autoantibodies with duration of CFS illness. Among CFS subjects in both study populations, those who had been sick longer had higher rates of autoantibodies than those that report shorter duration of illness. MAP2 is a neuron specific cytoskeleton protein. Autoantibodies to MAP2 have been demonstrated in patients with neuropsychiatric systemic lupus erythematosus [14]. While no lesions or loss of central nervous system function is reported in people with CFS, loss of memory, concentration and cognitive impairment are common complaints. Future studies will attempt to associate assessment of these parameters with the presence of MAP2 autoantibodies.

## Conclusion

There was no evidence of higher rates of the common autoantibodies in people with CFS. However, certain subsets of CFS subjects that had higher rates of antibodies to microtubule-associated protein 2 (MAP2) and ssDNA. Autoantibodies to specific host cell antigens may be a useful approach to stratify CFS subjects and provide clues to CFS etiologies.

## Competing interests

The author(s) declare that they have no competing interests.

## Authors' contributions

SDV was instrumental in the design of the experimental approach, analysis, presentation, discussion of these data and manuscript preparation. WCR contributed to the design of the experimental approach, and to the manuscript preparation. Both authors read and approved the final manuscript.
